# Short-course Rifaximin therapy efficacy and lactulose hydrogen breath test in Chinese patients with diarrhea-predominant irritable bowel syndrome

**DOI:** 10.1186/s12876-020-01336-6

**Published:** 2020-06-12

**Authors:** Xiaojun Zhuang, Zhenyi Tian, Mei Luo, Lishou Xiong

**Affiliations:** Department of Gastroenterology and Hepatology, the First Affiliated Hospital of Sun Yat-Sen University, Guangzhou, 510080 China

**Keywords:** Irritable bowel syndrome, Rifaximin therapy, Small intestinal bacterial overgrowth, Quality of life

## Abstract

**Background:**

Gut microbiota alterations including small intestinal bacterial overgrowth (SIBO) might play a role in pathogenesis of irritable bowel syndrome (IBS). Rifaximin could effectively and safely improve IBS symptoms. The aim of this study was to investigate the effect of rifaximin on Gastrointestinal (GI) symptoms, quality of life (QOL) and SIBO eradication in Chinese IBS-D patients.

**Methods:**

This study included 78 IBS-D patients defined by the Rome IV criteria. Patients received 400 mg rifaximin twice daily for 2 weeks and 10-week follow-up. GI symptoms were assessed at week 0, 2, 4, 8 and 12. QOL and lactulose hydrogen breath test (LHBT) results were estimated at week 0 and 4.

**Results:**

All participants showed significant improvements in GI symptom subdomains after rifaximin treatment (all *P* < 0.05), which could maintain at least 10 weeks of follow-up. Additionally, QOL scores were increased with concomitant improvement of clinical symptoms (all *P* < 0.05). The 45 rifaximin-responsive patients (57.7%) achieved significantly greater GI-symptom improvement than non-responders (all *P* < 0.05). No GI symptoms were associated with SIBO (all *P* > 0.05). SIBO normalization after rifaximin treatment measured by LHBT was found in 44.4% (20/45) of patients with SIBO before treatment.

**Conclusion:**

A short course (2 weeks) of rifaximin improved GI symptoms and QOL in Chinese IBS-D patients whether they had SIBO or not. However, the efficacy of rifaximin could not be explained by the successful eradication of SIBO. Further studies on the therapeutic mechanisms of rifaximin in IBS are urgently needed.

## Background

Irritable bowel syndrome (IBS) is one of the most common functional bowel disorders, with a relapsing and remitting natural history characterized by abdominal pain that is associated with defecation or alterations in bowel habits [1]. The prevalence of IBS around the world is approximately 7–21%; it is 1–16% in China, but the prevalence differs depending on regions and diagnostic criteria [2, 3]. Dissatisfaction and comorbidities of traditional treatment are associated with a significant reduction in the quality of life (QOL) and growing social, sanitary and economic burden worldwide [4–6]. Patients are stratified into four subtypes based on the predominant bowel habit: constipation-predominant IBS (IBS-C), diarrhea-predominant IBS (IBS-D), mixed IBS (IBS-M) and unclassified IBS (IBS-U) [1]. Although the precise etiology of IBS remains unknown, the possible mechanisms include visceral hypersensitivity, gut motility dysfunction, immunomodulation disturbances, gut microbiota alterations and an imbalance in brain-gut axis interactions [7–10]. In addition, new-onset IBS symptoms following acute infectious gastroenteritis might also suggest a microbial pathogenesis for IBS [11].

Alterations in the quantity or composition of the gut microbiota with subsequent metabolic disturbances have been observed in patients with IBS. In a recent systematic review, increased abundances of *Enterobacteriaceae* and *Lactobacillaceae* at the family level and *Bacteroides* at the genus level were found in patients with IBS compared with controls, whereas the abundance of the order uncultured *Clostridiales I*, and the genera *Faecalibacterium* and *Bifidobacterium* were decreased in IBS patients [10]. Moreover, we previously reported alterations in the abundance of predominant fermenting bacteria involved in the pathophysiology of IBS-D (such as *Bacteroidales* and *Clostridiales*) [12]. Furthermore, an association between IBS and small intestinal bacterial overgrowth (SIBO) has been observed in some patients with IBS, although the causal relationship between SIBO and IBS remains to be elucidated [13–18]. SIBO might partly explain IBS symptoms, such as bloating, abdominal pain and changes in bowel habits. A definite diagnosis of SIBO is characterized by greater than 10^5^ microorganisms/ml with poly-microbial flora in cultures of duodenal or jejunal fluid [19]. However, SIBO is diagnosed by various breath tests clinically, and the lactulose hydrogen breath testing (LHBT) is most commonly used, as intestinal samples are difficult to obtain [20–22]. Gut microbiota alterations indicate that the manipulation of the composition of the intestinal microbiota with probiotics, prebiotics, antibiotics, dietary interventions and fecal microbiota transplantation may be useful treatment approaches [23].

Rifaximin, as a gastrointestinal (GI)-specific broad-spectrum antibiotic, shows activity against both gram-positive and gram-negative, anaerobic and aerobic bacteria [24]. Since it displays low systemic absorption and no clinically significant interactions with other drugs, rifaximin may be a promising treatment for IBS, mainly due to its ability to act on IBS pathogenesis by modulating gut microbiota, altering bacterial metabolism, preserving epithelial function and reducing proinflammatory cytokine production [25–27]. Additionally, prior studies on rifaximin in nonconstipated IBS patients with SIBO indicated that rifaximin treatment is effective in improving IBS symptoms and eradicating SIBO [28–32]. However, there are few studies on the association of GI symptoms and QOL with LHBT results in the Chinese population.

The overall aim of this study was to explore whether rifaximin treatment improves GI symptoms (abdominal discomfort, abdominal distension, abdominal pain, defecatory urgency, diarrhea and incomplete evacuation) and QOL (physical functioning, role-physical, bodily pain, general health, vitality, social functioning, role-emotional and mental health) in Chinese IBS patients. We hypothesized that rifaximin treatment could relieve GI symptoms and optimize QOL by normalizing SIBO a measured by the LHBT.

## Methods

### Ethics statement

This study was conducted at the Department of Gastroenterology and Hepatology, the First Affiliated Hospital of Sun Yat-sen University, from December 2016 to December 2018. The protocol was approved by the Medical Ethics Committee of the First Affiliated Hospital of Sun Yat-sen University, and all patients provided written informed consent. The ClinicalTrials.gov ID for the study is NCT02565654.

### Study subjects

Seventy-eight patients with IBS-D were recruited into this study by two gastroenterologists with expertise in IBS. The inclusion criteria were men or women aged 18 years and above who met the Rome IV criteria for IBS-D, symptoms for more than 6 months, and patients with IBS symptoms as mentioned and normal appearance of the gastrointestinal mucosa. The exclusion criteria were clinical evidence of inflammatory bowel disease, a history of duodenal or gastric ulcers, diverticulitis or infectious gastroenteritis, abdominal surgery, cardiac, pulmonary, hepatic, renal or metabolic disease, use of antibiotics, probiotics, prebiotics, corticosteroids, proton-pump inhibitors, or IBS prescription medications within the last 4 weeks. A colonoscopy was performed on all patients to rule out organic disease.

### Study design and procedures

All participants received 400 mg rifaximin (Xifaxan®, ALFASIGMA S.p.A., Bologna, Italy) twice daily for 2 weeks. Then, they were further followed-up for an additional 10 weeks after treatment cessation. For recruited patients, they were informed to not to take any other prebiotics, probiotics and antibiotic but rifaximin throughout the observation period. All investigators were asked to complete GI symptom questionnaire and an IBS-relevant QOL questionnaire based on the Medical Outcomes Study (MOS) item short-form health survey (SF-36). The symptoms were recorded in a diary at baseline, the end of the treatment (week 2), end of the 2-week follow-up (week 4), end of the 6-week follow-up (week 8), and the end of the 10-week follow-up (week 12). The assessed symptoms were abdominal discomfort, abdominal distension, abdominal pain, diarrhea, defecatory urgency and incomplete evacuation; the severity of GI symptoms was rated using a 7-point Likert scale (0 = not at all, 1 = hardly, 2 = somewhat, 3 = moderately, 4 = a good deal, 5 = a great deal, and 6 = a very great deal). In addition, a QOL questionnaire was completed by IBS-D patients at baseline and at the end of the 2-week posttreatment period. The SF-36 is a 36-item questionnaire that measures 8 domains relevant to patients with IBS: (1) Physical Functioning, (2) Role-physical, (3) Bodily pain, (4) General Health, (5) Vitality, (6) Social Functioning, (7) Role-Emotional, and (8) Mental Health. Finally, all patients received an LHBT at baseline and the end of 2 weeks after rifaximin treatment.

### Evaluation of SIBO by LHBT

The LHBT was performed according to a standard protocol. Patients did not receive any antibiotics, probiotics, prebiotics, or laxatives in the 4 weeks preceding the test. To minimize basal H_2_ excretion, IBS-D patients were asked to avoid foods containing complex carbohydrates (bread, potato, and corn) and fiber in the previous evening and fasted for at least 12 h before the breath test. Cigarette smoking and physical exercise were not allowed for 2 h before and during the test. On the day of testing, patients washed their mouths with 20 ml of 0.05% chlorhexidine (Koutai, Shenzhen, China) to eliminate the fermentation by oropharyngeal bacteria flora. LHBT was performed in IBS-D patients using a gas analyzer (GastroLyzer R Breath Hydrogen Monitor; Bedfont Science Ltd., UK). Immediately before the test, a sample of expired air was taken to assess the basal H_2_ concentration. Then, 10 g of lactulose dissolved in 100 ml of water was administered within 30 s, and the expired air was sampled every 30 min over the next 3 consecutive hours by a trained study coordinator.

According to the literature and our previous results [12, 33, 34], LHBT was considered indicative of the presence of SIBO when (i) a baseline value of H_2_ ≥ 20 ppm and/or (ii) a > 20 ppm increase in H_2_ over basal values occurred within 90 min of lactulose administration.

### Outcome evaluation

The primary endpoint was to assess the improvement in GI symptoms and QOL after 2 weeks of rifaximin treatment in the Chinese population. The secondary endpoint was to compare the LHBT results before and after treatment with rifaximin. We also explored the response rate to rifaximin treatment by analyzing the self-reported GI symptoms, and the response to treatment was defined as a more than 50% improvement in the global GI symptoms 2 weeks after the cessation of treatment. Finally, we sought to search for symptoms closely associated with SIBO.

### Statistical analysis

All statistical analyses were performed using SPSS version 23.0 (SPSS, Inc., Chicago, IL, United States) and Graph Prism version 7.0 (GraphPad Software, Inc., La Jolla, CA, United States). Continuous data were analyzed using Student’s t-test or Mann-Whitney U-test where appropriate. Categorical data were analyzed using a chi-square test. Pearson correlation coefficient analysis was used to assess the relationship between GI symptoms and SIBO. All tests for significance were two-sided and *P* < 0.05 was considered statistically significant.

## Results

### Demographic and clinical characteristics of patients

Seventy-eight patients (33.5 years [18–58], 52 [66.7%] male) with IBS-D were enrolled in this study, and all participants completed a 12-week follow-up. Though IBS-D patients were more often male, the difference in age between the LHBT-positive and LHBT-negative groups was nonsignificant. Table 1 summarizes the demographic and clinical characteristics of all IBS-D patients. At baseline, 45 patients (29/16) with SIBO were younger than those (23/10) with a negative LHBT result (32.13 ± 7.48 vs 37.24 ± 9.95, *P* = 0.016). In addition, there was no significant difference in the GI symptoms and QOL scores between the LHBT-negative and LHBT-positive groups. Moreover, no GI symptoms were found to be associated with the presence of SIBO (Table 2).

**Table 1:** Demographic and clinical characteristics of all included patients at baseline

Clinical factors	LHBT (+)	LHBT (−)	*P* value
Age (mean, years)	32.13 ± 7.48	37.24 ± 9.95	0.016
Gender (M/F)	29/16	23/10	0.627
GI symptoms (mean)	17.04 ± 5.02	17.42 ± 4.40	0.724
Abdominal discomfort	2.31 ± 1.38	2.55 ± 1.23	0.432
Abdominal distension	1.84 ± 1.35	2.00 ± 1.17	0.589
Abdominal pain	3.07 ± 1.37	2.45 ± 1.37	0.056
Defecatory urgency	3.78 ± 1.31	3.79 ± 1.29	0.973
Diarrhea	4.11 ± 3.56	3.64 ± 1.06	0.460
Incomplete evacuation	2.38 ± 1.35	3.00 ± 1.39	0.053
Quality of life (mean)	506.97 ± 126.70	477.82 ± 105.95	0.273
Physical Functioning	94.78 ± 11.03	93.03 ± 6.49	0.384
Role-physical	61.11 ± 40.68	52.27 ± 40.68	0.347
Bodily pain	57.72 ± 20.48	57.27 ± 21.06	0.449
General Health	43.33 ± 19.86	43.55 ± 16.13	0.958
Vitality	57.11 ± 16.57	55.91 ± 19.86	0.778
Social Functioning	75.67 ± 20.94	68.32 ± 21.79	0.138
Role-Emotional	55.55 ± 42.05	44.44 ± 37.89	0.226
Mental Health	61.69 ± 16.79	63.03 ± 13.42	0.696

**Table 2:** Correlation analysis between SIBO and GI symptoms

	SIBO	
	ρ	*P* value
Abdominal discomfort	0.081	0.483
Abdominal distension	0.083	0.468
Abdominal pain	0.231	0.052
Defecatory urgency	0.013	0.909
Diarrhea	0.064	0.578
Incomplete evacuation	0.199	0.081

Note: ρ, Spearman rank correlation coefficient; SIBO, small intestinal bacterial overgrowth;

GI symptoms, gastrointestinal symptoms

### Effect of rifaximin on SIBO

For the subjects with SIBO before treatment, 25 (44.4%) had a negative LHBT after 2 weeks of rifaximin treatment (week 4). Furthermore, patients who received rifaximin treatment more often tended to have a negative LHBT (45/33 [42.3%] vs 25/53 [67.9%], *P* = 0.001) and had reduced hydrogen production. As shown in Table 3, there was significant difference in SIBO rate of Chinese IBS-D patients before and after rifaximin treatment (57.7, 95% CI, 46.5–68.9% vs 32.1, 95% CI: 21.5–42.6%; *P* <0.001). In addition, there was no significant difference in age and gender between patients with and without LHBT normalization after 2 weeks of rifaximin therapy.

**Table 3:** SIBO rate in Chinese IBS-D patients pre- and post- rifaximin treatment

	LHBT		95% CI (%)	*P* value
	Positive/N (%)	Negative/N (%)		
Pre-treatment	45 (57.7)	33 (42.3)	46.5–68.9	<0.001
Post-treatment	25 (32.1)	53 (67.9)	21.5–42.6	

Note: *SIBO*, Small intestinal bacterial overgrowth; *IBS*, Irritable bowel syndrome; *IBS-D*, diarrhea-predominant IBS

### Effect of rifaximin on GI symptoms

**Fig. 1:**
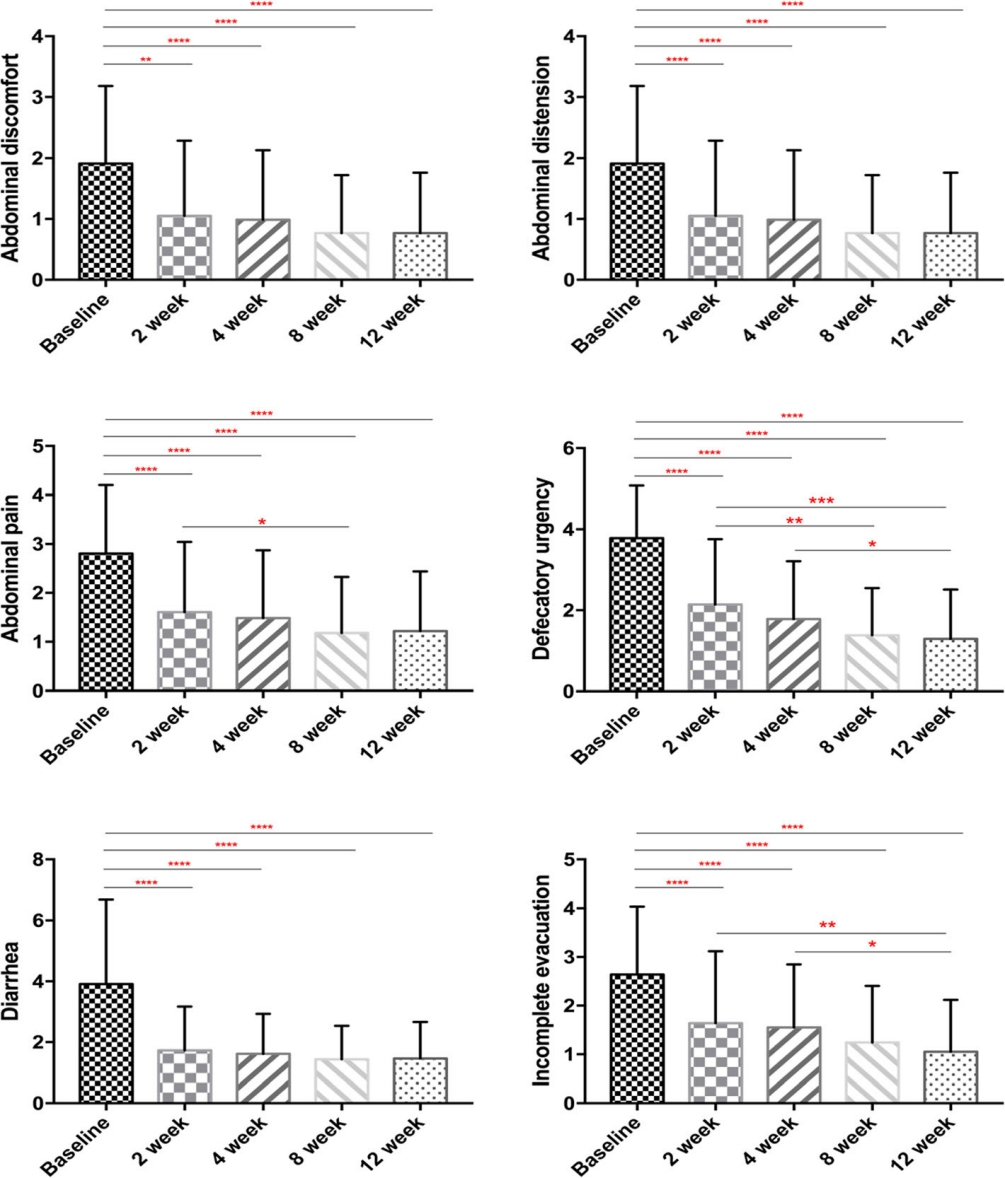
GI symptoms scores in IBS-D patients at different times during the study. Note: *P < 0.05, ** *P* < 0.01, *** *P* < 0.001; IBS, irritable bowel syndrome; IBS-D, diarrhea-predominant IBS; GI symptoms, gastrointestinal symptoms

A symptomatic evaluation after 2 weeks of rifaximin treatment might show improvements before the LHBT normalizes (Fig. 1). The IBS symptoms of abdominal pain, abdominal discomfort, abdominal distension, diarrhea, defecatory urgency and incomplete evacuation improved significantly after rifaximin treatment, and the symptom relief persisted for at least 10 weeks during the follow-up period (all *P* < 0.05). In addition, 45 (57.7%) patients experienced a clinical response accompanied by a global IBS symptoms score reduction of at least 50% (5.36 ± 3.27 vs 13.79 ± 5.21, *P* < 0.001). The response group reported a full recovery or greater improvement in their symptoms than the nonresponse group, showing a significant difference in every GI symptom (Table 4). However, the difference in age and gender between the response and nonresponse groups was not statistically significant. For the IBS-D patients with SIBO, the GI symptoms showed significant improvement in each of the six symptom scores and in the global score after SIBO eradication through rifaximin treatment. Nevertheless, subjects without SIBO eradication exhibited a similar resolution in five GI symptoms, but not abdominal discomfort, suggesting that the effect of rifaximin in IBS-D is not explained by SIBO eradication. In contrast, LHBT-negative patients at baseline showed significant improvement in each of the six GI symptoms after treatment. Finally, IBS-D patients with SIBO or without SIBO at week 4 recorded similar GI symptoms scores, regardless of whether they succeeded in eradicating SIBO (Table 5).

**Table 4:** Comparison between the Response and Non-Response Groups after rifaximin treatment at week 4

Clinical factors	Response group (*n* = 45)	Non-response group (*n* = 33)	*P* value
Age (mean, years)	34.82 ± 9.04	33.58 ± 8.84	0.544
Gender (M/F)	31/14	21/12	0.627
LHBT (+/−)	18/27	8/25	0.145
GI symptoms (mean)	5.36 ± 3.27	13.79 ± 5.21	< 0.001
Abdominal discomfort	0.87 ± 0.89	2.45 ± 1.23	< 0.001
Abdominal distension	0.49 ± 0.70	1.67 ± 1.29	< 0.001
Abdominal pain	0.91 ± 0.93	2.27 ± 1.53	< 0.001
Defecatory urgency	1.09 ± 1.13	2.73 ± 1.26	< 0.001
Diarrhea	1.02 ± 0.97	2.42 ± 1.23	< 0.001
Incomplete evacuation	0.98 ± 1.03	2.33 ± 1.22	< 0.001
Quality of life (mean)	616.34 ± 84.06	545.34 ± 94.70	0.001
Physical Functioning	97.33 ± 4.47	94.85 ± 5.93	0.048
Role-physical	82.78 ± 24.90	67.42 ± 36.70	0.031
Bodily pain	77.34 ± 13.70	62.36 ± 19.88	< 0.001
General Health	59.22 ± 16.52	45.64 ± 16.70	0.001
Vitality	67.33 ± 15.06	61.67 ± 14.23	0.094
Social Functioning	85.63 ± 10.52	76.67 ± 19.98	0.012
Role-Emotional	76.30 ± 32.28	72.73 ± 30.57	0.621
Mental Health	70.40 ± 16.30	64.00 ± 18.08	0.112

**Table 5:** GI symptoms and QOL comparisons between IBS-D patients with and without SIBO eradication after rifaximin treatment

Clinical factors	LHBT (+)	LHBT (−)	*P* value
GI symptoms (mean)	7.88 ± 6.15	8.95 ± 5.23	0.531
Abdominal discomfort	1.64 ± 1.29	1.20 ± 1.20	0.243
Abdominal distension	0.92 ± 1.04	0.90 ± 1.29	0.956
Abdominal pain	1.64 ± 1.35	1.45 ± 1.28	0.631
Defecatory urgency	1.32 ± 1.44	2.05 ± 1.28	0.078
Diarrhea	1.20 ± 1.29	1.95 ± 1.10	0.051
Incomplete evacuation	1.16 ± 1.11	1.55 ± 1.15	0.256
Quality of life (mean)	579.93 ± 106.11	599.05 ± 88.29	0.513
Physical Functioning	96.20 ± 4.63	98.25 ± 3.73	0.107
Role-physical	70.00 ± 35.36	81.25 ± 25.49	0.222
Bodily pain	71.92 ± 14.29	73.68 ± 15.41	0.697
General Health	53.60 ± 19.72	53.85 ± 16.69	0.963
Vitality	63.80 ± 14.74	64.00 ± 15.01	0.965
Social Functioning	84.57 ± 12.92	82.95 ± 13.41	0.685
Role-Emotional	72.00 ± 32.89	81.67 ± 33.29	0.336
Mental Health	67.84 ± 17.42	63.40 ± 18.55	0.417

Note: IBS, irritable bowel syndrome; IBS-D, diarrhea-predominant IBS; GI symptoms, gastrointestinal symptoms; QOL, quality of life; SIBO, small intestinal bacterial overgrowth

### Effect of rifaximin on the QOL

**Fig. 2:**
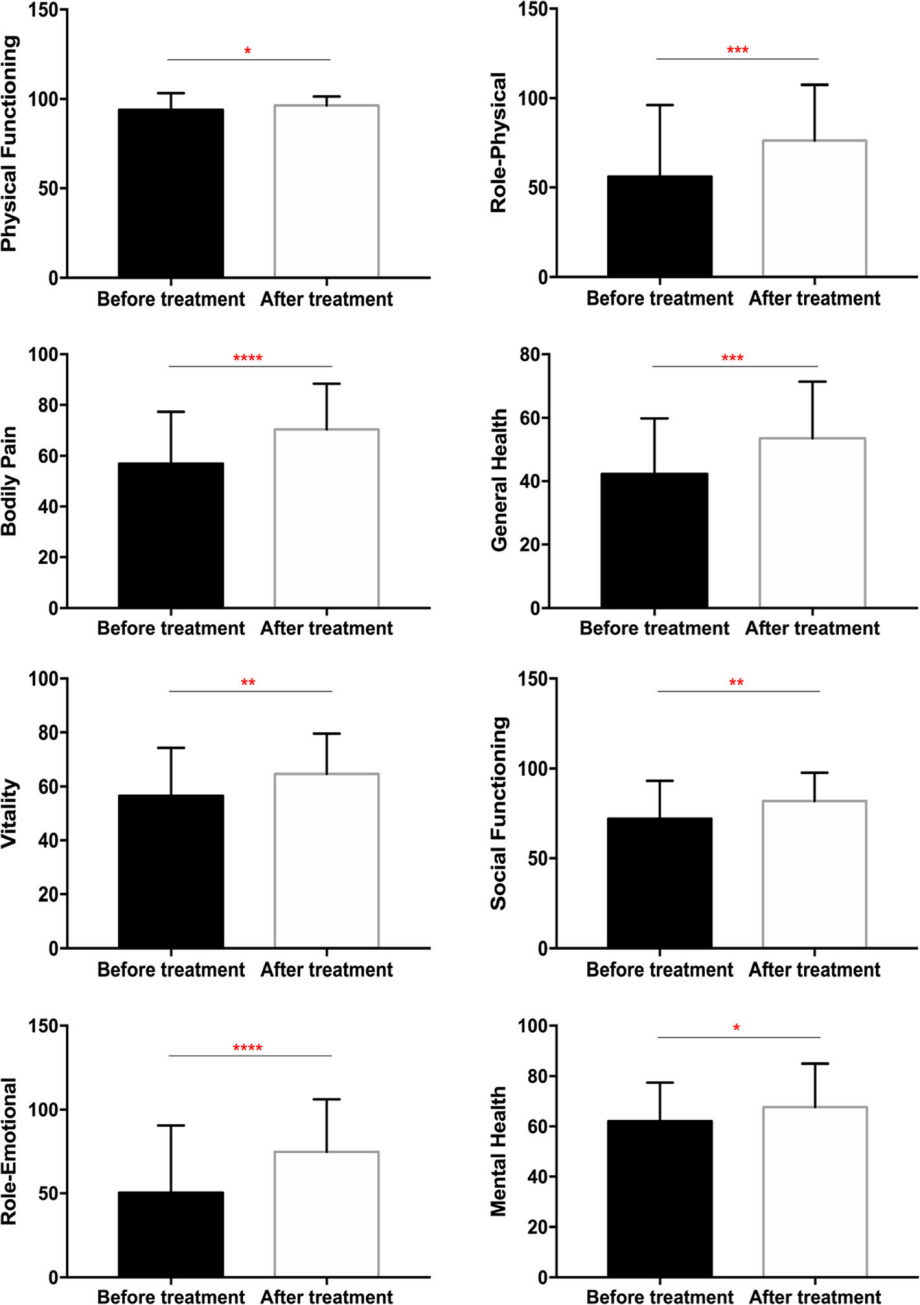
QOL scores comparison in IBS-D patients pre- and post-rifaximin treatment. Note: *P < 0.05, ** *P* < 0.01, *** *P* < 0.001; IBS, irritable bowel syndrome; IBS-D, diarrhea-predominant IBS; QOL, Quality of life

At baseline, all participants reported severely reduced QOL scores. Fortunately, total QOL scores significantly increased 2 weeks after the completion of treatment (week 4), indicating QOL improvement (Fig. 2). Compared to the nonresponse group, the response group reported significant alterations in five domains of QOL, with no significant difference in vitality, role-emotional and mental health (Table 4). Additionally, there was no significant difference in any of the eight domain scores or global QOL score between LHBT-positive and LHBT-negative groups. For the LHBT-positive IBS-D patients at baseline, bodily pain and general health improved significantly regardless of whether SIBO was successfully eliminated after rifaximin treatment. In contrast, a significant increase in seven QOL domain scores was observed in LHBT-negative patients at baseline with no significant difference in physical functioning after 2 weeks of rifaximin treatment. Finally, IBS-D patients with SIBO or without SIBO at week 4 recorded similar QOL scores, regardless of whether they succeeded in eradicating SIBO (Table 5).

### Adverse events

No patient developed any adverse events during rifaximin administration, except for two patients who reported transient nausea during rifaximin treatment. Overall, the treatment was well tolerated.

## Discussion

The findings of this study suggest that a short course (2 weeks) of rifaximin therapy is safe and efficacious for the treatment of IBS-D patients as assessed using the ROME IV criteria. The GI symptom relief, QOL improvement and SIBO normalization after rifaximin treatment observed in our study imply that rifaximin is an effective option for the treatment of IBS-D. Furthermore, the effectiveness of the short-course rifaximin treatment was sustained for at least 12 weeks after treatment. To our knowledge, this is the first study evaluating the effect of rifaximin on GI symptoms and QOL based on SIBO in Chinese patients with IBS-D.

As previously stated, the etiological and symptomatic manifestation of IBS and SIBO may overlap, and SIBO has been postulated to be a pathophysiological mechanism for IBS. Moreover, SIBO is in fact associated with IBS-like symptoms, such as bloating, abdominal pain, and a change in bowel habits. The frequency of SIBO among IBS patients ranges between 4 and 78%, and the variations in prevalence of SIBO in previous studies might be attributable to differences in the geographical origins of the studied populations, different criteria for the diagnosis of IBS and methods for the diagnosis of SIBO using different breath tests [33–35]. The response to rifaximin treatment in IBS-D patients has been shown to correlate with the normalization of the LHBT results [32, 36, 37]. In our study, 57.7% of the included patients had a positive LHBT, and 20 showed LHBT normalization after 2 weeks of rifaximin treatment, with a SIBO eradication rate of 44.4%. In addition, the LHBT-positive subjects were younger than the LHBT-negative subjects. In contrast to our study, a recent meta-analysis involving 32 studies reported that the overall eradication rate according to an intention-to-treat analysis was 70.8% (95% CI: 61.4–78.2; I^2^ = 89.4%) and according to a per-protocol analysis was 72.9% (95% CI: 65.5–79.8; I^2^ = 87.5%) [32]. However, another meta-analysis of eight studies showed that the overall breath-test normalization rate with rifaximin was 49.5%, which is somewhat similar to the result of our study [38]. The marked discrepancy in rates of SIBO eradication might be related to geographical, dietary or ethnicity differences in the microbiomes of the study populations or the dose of rifaximin. The findings of our study suggest that either SIBO plays a limited role in causing IBS-associated gut microbiota disturbances or that LHBT is not a good test to measure SIBO.

There was no significant difference in GI symptoms and QOL scores between the LHBT-positive and LHBT-negative groups. After 2 weeks of rifaximin treatment, all individual and global symptoms displayed instant improvement, and these effects lasted for at least 10 weeks during the follow-up period. However, not all patients showed a desirable response to rifaximin therapy according to the formal prespecified criteria for a response. In the LHBT-positive group, the response rate was relatively high (40.0%) compared with that in the LHBT-negative group (24.2%), but the difference was not statistically significant. More importantly, participants with LHBT normalization after treatment appeared to experience symptomatic improvement in all of the six symptoms, whereas those without SIBO eradication showed similar symptom relief, with the exception of abdominal discomfort. However, more severe diarrhea was recorded in subjects with LHBT normalization than those without LHBT normalization, which means that this nonabsorbable antimicrobial agent did not completely reverse the gut microflora dysbiosis when eradicating SIBO. In addition, subjects with a negative LHBT at baseline also achieved individual and global GI symptom improvements that persisted after rifaximin intervention. In the well-known TARGET 1 and TARGET II studies, only 40% of patients responded to rifaximin, but treatment with rifaximin for 2 weeks provided significant relief of IBS symptoms including bloating, abdominal pain, and loose or watery stools [28]. The inconsistent response to rifaximin in various studies may be due to IBS heterogeneity, and LHBT normalization might not be a good marker to assess the response to rifaximin. In contrast to our study, an open-label study from Europe reported an improvement in individual symptoms (abdominal pain, diarrhea, and bloating) as well global symptoms with 800 mg/day rifaximin for 2 weeks [39]. Recently, a study of retreatment with rifaximin showed a 33% response rate in the rifaximin group compared to 25% in the placebo group (*P* = 0.02), consistent with FDA guidelines for the clinical assessment of IBS drugs in the TARGET 3 study [40].

At baseline, all participants reported reduced QOL scores. Interestingly, the IBS-QOL overall and all subdomain scores improved from baseline for up to 2 weeks posttreatment and were accompanied by symptom relief in the included patients. Indeed, responders had a significantly greater improvement in the overall QOL score than nonresponders, which implies that a sufficient improvement in patient clinical symptoms guarantees that their QOL improves. Furthermore, rifaximin treatment significantly impacted bodily pain and general health in patients with a positive LHBT, regardless of whether SIBO was successfully eradicated. Interestingly, our findings indicate that treatment with rifaximin favorably improves the total QOL and seven subdomain scores in LHBT-negative patients with IBS-D, which is consistent with previously reported data [41]. Similar effects have been seen in another study, the findings of which suggested that the increased improvement in QOL following repeat treatment with rifaximin is associated with a reduced chance of subsequent symptom relapse [42]. However, rifaximin was not effective in improving IBS symptoms and QOL in Gulf War veterans with non-constipated IBS [43]. Finally, LHBT-positive and LHBT-negative IBS-D patients did not differ significantly in their reported post rifaximin total QOL or subscale scores.

Rifaximin was approved by the US Food and Drug Administration in 2015 to treat adults with IBS-D [44]. Although the mechanism of action of rifaximin in IBS is complex, a leading hypothesis proposes that rifaximin modulates intestinal flora imbalances. Mounting evidence has shown that rifaximin treatment induces alterations in the abundance of specific bacterial populations rather than affecting the overall composition of the microbiota in the treated subjects and has no apparent detrimental effects on gut microbiota [45–47]. On the one hand, rifaximin shows a potent killing effect on common SIBO pathogens [48, 49]. On the other hand, rifaximin appears to increase the abundance of certain potentially beneficial bacteria, such as *Faecalibacterium prausnitzii*, but reduces the abundance of detrimental bacteria such as *Clostridium*. In addition to the direct antibiotic effects of rifaximin on gut microbiota, rifaximin impacts the function of the gut microbiota (i.e., metabolism, adherence and virulence) [50–52]. Alterations in certain lipid species, saturated and unsaturated fatty acids, and products of carbohydrate metabolism were found in several studies focused on rifaximin treatment for IBS; these alterations might have beneficial effects on various symptoms (improved barrier function of the small bowel and reduced visceral hyperalgesia) of GI-related disease. For example, a study from Bajaj et al. found that alteration of gut bacterial linkages with metabolites rather than significant change in microbial abundance after rifaximin therapy, which especially linked to ammonia, aromatic amino acids and oxidative stress [50]. Furthermore, rifaximin could inhibit bacterial interactions with the host to reduce detrimental bacterial colonization, infection and the activation of the host immune response to prevent mucosal inflammation by reducing the level of proinflammatory mediators [53]. In addition, rifaximin is able to reduce bacterial virulence and translocation, has anti-inflammatory properties by increasing the relative abundance of *Faecalibacterium prausnitzi* endowed with powerful anti-inflammatory activities [54]. Taken together, these results show that the beneficial effects and safety of rifaximin treatment might be partly accounted for by resetting the gut microenvironment and modulating the inflammatory environment.

There are several limitations in this study. It was conducted in a single center with a relatively small sample size and open-label design so that conclusions should be drawn cautiously. Further limitations of our study are no control group and the lack of randomization. Additionally, the validity and interpretation of the LHBT for the diagnosis of SIBO is an ongoing controversy. The greatest weakness of the study is that the potential mechanisms by which rifaximin beneficially affects IBS-D patients with definite SIBO were not elaborated comprehensively. Nevertheless, it might be noteworthy that this is the first study to show that short-course rifaximin therapy is an appropriate treatment option for Chinese IBS-D patients.

## Conclusion

In conclusion, a short course of rifaximin treatment significantly improved the GI symptoms and QOL of Chinese IBS-D patients in this study, and 2-week rifaximin treatment led to the sustained improvement of IBS symptoms for at least 10 weeks, which is consistent with multiple previous large clinical trials of single and repeat treatment cycles. However, the efficacy of rifaximin could not be explained by the successful eradication of SIBO. More therapeutic mechanisms of rifaximin for IBS-D patients are warranted in further studies.

## Data Availability

All data and materials are not available in this study, and are available from the corresponding author on reasonable requests.

## Abbreviations

IBS: Irritable bowel syndrome
IBS-D: patients with diarrhea-predominant IBS
SIBO: Small intestinal bacterial overgrowth
LHBT: Lactulose hydrogen breath test
QOL: Quality of life
